# Supplementary material for: Resolving the full spectrum of human genome variation using Linked-Reads
Source: Genome Res. 2019 Apr;29(4):635–45. doi: 10.1101/gr.234443.118 (PMC6442396; doi:10.1101/gr.234443.118)
Supplement: Supplemental Material [file supp_gr.234443.118_Supplemental_longranger-2.2.zip › longranger-2.2.tar.bz2!/lr2.2-public/lib/stan/cmdstan/stan/doc/api/html/classstan_1_1gm_1_1arg__method.html]

Stan: stan::gm::arg\_method Class Reference


|  |  |
| --- | --- |
|  | Stan  2.5.0  probability, sampling & optimization |


- Main Page
- Related Pages
- Namespaces
- Classes
- Files

- Class List
- Class Index
- Class Hierarchy
- Class Members

All Classes Namespaces Files Functions Variables Typedefs Enumerations Enumerator Friends Macros Pages

- stan
- gm
- arg\_method

Public Member Functions |
List of all members

stan::gm::arg\_method Class Reference

`#include <arg_method.hpp>`

Inheritance diagram for stan::gm::arg\_method:

|  |  |
| --- | --- |
| Public Member Functions | |
|  | arg\_method () |
|  | |
| Public Member Functions inherited from stan::gm::list\_argument | |
|  | list\_argument () |
|  | |
|  | ~list\_argument () |
|  | |
| void | print (std::ostream \*s, int depth, const std::string prefix) |
|  | |
| void | print\_help (std::ostream \*s, int depth, bool recurse) |
|  | |
| bool | parse\_args (std::vector< std::string > &args, std::ostream \*out, std::ostream \*err, bool &help\_flag) |
|  | |
| virtual void | probe\_args (argument \*base\_arg, std::stringstream &s) |
|  | |
| void | find\_arg (std::string name, std::string prefix, std::vector< std::string > &valid\_paths) |
|  | |
| bool | valid\_value (std::string name) |
|  | |
| argument \* | arg (std::string name) |
|  | |
| std::vector< argument \* > & | values () |
|  | |
| std::string | value () |
|  | |
| std::string | print\_value () |
|  | |
| std::string | print\_valid () |
|  | |
| bool | is\_default () |
|  | |
| Public Member Functions inherited from stan::gm::argument | |
|  | argument () |
|  | |
|  | argument (const std::string name) |
|  | |
| virtual | ~argument () |
|  | |
| std::string | name () const |
|  | |
| std::string | description () const |
|  | |
| int | compute\_indent (const int depth) |
|  | |

|  |  |
| --- | --- |
| Additional Inherited Members | |
| Static Public Member Functions inherited from stan::gm::argument | |
| static void | split\_arg (const std::string &arg, std::string &name, std::string &value) |
|  | |
| Protected Attributes inherited from stan::gm::list\_argument | |
| int | \_cursor |
|  | |
| int | \_default\_cursor |
|  | |
| std::vector< argument \* > | \_values |
|  | |
| Protected Attributes inherited from stan::gm::valued\_argument | |
| std::string | \_default |
|  | |
| std::string | \_value\_type |
|  | |
| Protected Attributes inherited from stan::gm::argument | |
| std::string | \_name |
|  | |
| std::string | \_description |
|  | |
| int | indent\_width |
|  | |
| int | help\_width |
|  | |

## Detailed Description

Definition at line 14 of file arg\_method.hpp.

## Constructor & Destructor Documentation

|  |  |  |  |  |
| --- | --- | --- | --- | --- |
| stan::gm::arg\_method::arg\_method | ( |  | ) |  |

inline

Definition at line 18 of file arg\_method.hpp.

---

The documentation for this class was generated from the following file:

- src/stan/gm/arguments/arg\_method.hpp


---

[ Stan Home Page ]

*© 2011–2014,
Stan Development Team.*
